# Low plasmatic concentration of intensified antiretroviral therapy in a pregnant woman: a case report

**DOI:** 10.1186/s13256-019-2148-y

**Published:** 2019-07-23

**Authors:** Sheila Chiesi, Sebastiano Rizzardo, Daniela Piacentini, Giorgia Be, Emanuela Lattuada, Evelina Tacconelli, Massimiliano Lanzafame

**Affiliations:** Infectious Diseases Department, University Hospital of Verona, Piazzale L.A. Scuro, Verona, Italy

**Keywords:** HIV-1, Pregnancy, Pharmacokinetics, C_trough_, Dolutegravir, Darunavir

## Abstract

**Background:**

Identifying the most appropriate antiretroviral regimen for pregnant women with Human Immunodeficiency Virus (HIV-1) infection can be challenging, mainly due to pregnancy-related physiological alterations which can significantly reduce maternal drug plasma concentration. We would like to report our experience as it consists of an unusual case of low plasmatic concentration of antiretroviral drugs despite regimen intensification in a HIV-positive pregnant woman. It also underlines the need for accurate monitoring and treatment adjustment in pregnant women with Human Immunodeficiency Virus (HIV).

**Case presentation:**

A 26-year-old Brazilian woman with HIV-1 infection attending our out-patient clinic presented with low plasmatic concentration of antiretroviral drugs and persistent detectable viral load despite regimen intensification during pregnancy.

Trough plasma concentrations of dolutegravir and darunavir were measured by validated liquid chromatography–mass spectrometry. At 23 weeks of gestation it showed a lower value than expected in non-pregnant adults, compared to a normal level of plasma concentration measured at 10 weeks after delivery. Our patient and the baby had no regimen-related adverse effects.

**Conclusions:**

Physiological changes during pregnancy can affect pharmacokinetics and reduce a mother’s bioavailability of antiretroviral drugs, potentially altering their pharmacological activity. A personalized treatment and a careful follow-up are hence mandatory for this key population.

## Background

Antiretroviral (ARV) treatment of human immunodeficiency virus (HIV) positive pregnant women has two main goals: to reduce maternal morbidity and mortality and to prevent mother-to-child HIV transmission [1]. These effective drugs have reduced the risk of HIV transmission from mother to infant to historically low levels both in the USA and in Europe [2]. The World Health Organization (WHO) recommends combination antiretroviral therapy (cART) for all pregnant women disregarding baseline CD4 count or viral load (http://www.who.int/hiv/pub/toolkits/keypopulations-2016-update/en). However, identifying the most appropriate regimen for this population can be challenging. The preferred ARV combination for pregnant women differs from the ARV combination for the general HIV-infected adult population, mainly because of altered pharmacokinetics during pregnancy. Pregnancy can in fact lead to significant reductions in ARV drugs bioavailability, particularly during the third trimester [3]. The main pregnancy-associated physiological alterations are induction of hepatic drug-metabolizing enzymes and reduced concentration of plasma proteins due to hemodilution [4]. For most nucleoside/nucleotide reverse transcriptase inhibitors and protease inhibitors, a decreased plasma concentration during pregnancy due to their metabolism by the cytochrome P450 3A enzyme family has been demonstrated [3]. On the other hand, integrase inhibitors follow a different metabolic pathway, which involves mainly UGT1A1, with minor contributions from CYP3A4 [5]. Induction of all of these enzymes may lower the bioavailability of ARV drugs during pregnancy, as reported by many authors [6, 7].

In order to minimize the likelihood of vertical transmission of the virus, some authors suggested intensifying the drug regimen during pregnancy by adding drugs or increasing their daily dose [8, 9], even if the cost/effectiveness of this strategy remains unclear.

## Case presentation

Here we report on a case of low plasmatic concentration of ARV drugs, with persistent detectable viral load despite regimen intensification, in a pregnant 26-year-old Brazilian woman with HIV-1 infection.

The first diagnosis of HIV was made in August 2016; at diagnosis, her CD4 count was 60 cells/mm^3^ and plasmatic viral load was 7,060,000 copies/mL. No drug resistance was reported at the baseline test. She did not report any alcohol or drug addiction, neither was she under any medical treatment. She was hence immediately started on ARV therapy. In accordance with the national Italian guidelines [10], a cART with abacavir/lamivudine/dolutegravir (DTG) (600/300/50 mg) once daily was introduced, which seemed to be well tolerated. Despite a significant reduction in the viral load (1650, 143, 58 copies/mL after 1, 2, and 4 months respectively), she never achieved plasmatic undetectability (HIV-ribonucliec acid (RNA) < 50 copies/mL) at follow-up. When asked, she reported poor adherence to the therapeutic regimen in spite of good tolerability.

In February 2017 she got pregnant. She was married and had adequate social and familial support and thus decided to go through with her pregnancy. The viral load at the beginning of pregnancy was 77 copies/mL with a CD4 count of 219 cells/mm^3^. After 1 month, the viral load was lower but still detectable (53 copies/mL). It was thus decided to implement the ongoing therapy with darunavir (DRV)-ritonavir (800/100 mg) once daily. Two months later, plasmatic HIV-RNA was 11,400 copies/mL in a context of reported good adherence. Furthermore, our patient did not report any flu-like illness or alterations of her general status which could have contributed to the increase in the viral load. Anticipating that bioavailability of DTG would be further reduced in the second and third trimesters, an additional dose of DTG (50 mg) was therefore included in the regimen. At the following control, 4 weeks later, plasmatic HIV-RNA was 252 copies/mL.

In order to maximize our patient’s adherence to the ARV therapy, the frequency of controls was increased to a monthly basis; when interviewed, she appeared motivated to adhere to the treatment and to follow any medical advice in order to preserve her health and the baby’s health. The actual intake of the pills was assessed by an electronic monitoring system of withdrawn drug boxes. In addition, at 23 weeks of gestation we collected a blood sample to determine the effective plasmatic concentration of DTG and DRV and to consequently better document plasmatic levels of ARV drugs. Trough plasma concentration (C_trough_) was measured by validated liquid chromatography–mass spectrometry (LC-MS/MS). For both drugs, analysis revealed a C_trough_ of 379 ng/mL (DTG) and 152 ng/mL (DRV), which were lower than expected in non-pregnant adults [11].

Along with controls at our HIV clinic, our patient underwent regular gynecologic follow-up. In addition to HIV infection, the physiology of her pregnancy was considered. The fetus showed normal growth and no major abnormalities were detected at ultrasound examinations.

At 34 weeks of gestation, the viral load dropped again to 81 copies/mL with a CD4 count of 177 cells/mm^3^; in accordance with the Italian guidelines [10], a cesarean section was thus recommended. Two weeks later, our patient delivered a healthy baby; there were no subsequent obstetric complications.

On review at 8 weeks, mother and baby were well; the baby’s T and B cell numbers were normal, and HIV-RNA was undetectable. She is thus far uninfected.

Ten weeks after delivery, DTG and DRV concentrations in maternal blood were again examined. The C_troughs_ were 3042 ng/mL (DTG) and 1748 ng/mL (DRV), showing a normal level of bioavailability for a non-pregnant adult [11]. Our patient’s regimen was then de-intensified to abacavir/lamivudine/DTG (600/300/50 mg) once daily. Her viral load declined to 38 copies/mL with a CD4 count of 248 cells/mm^3^ 1 month after treatment modification. After another 2 months, her CD4 cell count rose to 398 cells/mm^3^ and HIV-RNA was < 20 copies/ml.

## Discussion and conclusions

Preliminary results from an ongoing observational study in Botswana aroused some concerns on the use of DTG during pregnancy, especially at the time of conception or early in the first trimester, because of an apparent higher risk for neural tube defects in the infants [12]. Despite a recent warning both from the Food and Drug Administration (FDA) and the European Medicines Agency (EMA), a precautionary discouraging of the use of DTG in pregnancy or in women of childbearing age, a systematic review on the safety of this drug for pregnant women showed a risk of adverse birth outcomes and congenital abnormalities that was similar to results from historical control studies of HIV-positive women [13].

In our experience, DTG-containing regimens have always been well tolerated during pregnancy. In addition, no side effects in mothers or neural tube defects in babies born to women using DTG before or during pregnancy have been reported.

It is, however, true that persistent detectable viral load together with a low bioavailability of ARV drugs despite regimen intensification in pregnant women has been previously described [7]. Furthermore, some authors already expressed concerns over lower plasmatic concentrations of DTG during pregnancy, leading to an increase in the daily dosing [14]. We were also aware of the potential reduction of DRV concentration during pregnancy, as it has been shown by several studies of pharmacokinetics. A 800-mg DRV dose administered twice daily did not increase DRV exposure in pregnant women, thus the use of this increased twice daily dose during pregnancy is not recommended [9]. On the other hand, switching to DRV-ritonavir (600/100 mg) administered twice daily could have further compromised adherence.

Physiological changes during pregnancy can affect pharmacokinetics and reduce bioavailability of ARV drugs, potentially altering pharmacological activity. This pharmacological effect has been seen for DTG but also for other ARVs, with levels of plasmatic concentration maintained underneath effective levels.

As shown by this case report, plasmatic concentration of both DTG and DRV were significantly lower during the second trimester of pregnancy despite the increase in the daily dosing and reported good adherence. On the other hand, normal levels of the drugs, in association with a reduced viral load and a good count of CD4 were measured 5 weeks after delivery. Our experience confirms the need for a careful follow-up and adjustment of ongoing ARV treatment for pregnant women infected with HIV-1.

## Data Availability

Data sharing is not applicable to this article as no datasets were generated or analyzed during the current study.

## Abbreviations

ARV: Antiretroviral
cART: Combination antiretroviral therapy
C_trough_: Trough plasma concentration
DRV: Darunavir
DTG: Dolutegravir
EMA: European Medicines Agency
FDA: Food and Drug Administration
HIV: Human Immunodeficiency Virus
LC-MS: Liquid chromatography–mass spectrometry
RNA: Ribonucliec Acid
